# Organizational and community resilience for COVID‐19 and beyond: Leveraging a system for health and social services integration

**DOI:** 10.1111/1475-6773.14250

**Published:** 2023-10-16

**Authors:** Mark D. Fleming, Nadia Safaeinili, Margae Knox, Amanda L. Brewster

**Affiliations:** ^1^ School of Public Health University of California Berkeley Berkeley California USA

**Keywords:** community resilience, COVID‐19, organizational resilience, social determinants of health, system alignment

## Abstract

**Objective:**

To examine how a preexisting initiative to align health care, public health, and social services influenced COVID‐19 pandemic response.

**Data Sources and Study Setting:**

In‐depth interviews with administrators and frontline staff in health care, public health, and social services in Contra Costa County, California from October, 2020, to May, 2021.

**Study Design:**

Qualitative, semi‐structured interviews examined how COVID‐19 response used resources developed for system alignment prior to the pandemic.

**Data Collection:**

We interviewed 31 informants including 14 managers in public health, health care, or social services and 17 social needs case managers who coordinated services across these sectors on behalf of patients. An inductive‐deductive qualitative coding approach was used to systematically identify recurrent themes.

**Principal Findings:**

We identified four distinct components of the county's system alignment capabilities that supported COVID‐19 response, including (1) an organizational culture of adaptability fostered through earlier system alignment efforts, which included the ability and willingness to rapidly implement new organizational processes, (2) trusting relationships among organizations based on prior, positive experiences of cross‐sector collaboration, (3) capacity to monitor population health of historically marginalized community members, including information infrastructures, data analytics, and population monitoring and outreach, and (4) frontline staff with flexible skills to support health and social care who had built relationships with the highest risk community members.

**Conclusions:**

Prior investments in aligning systems provided unanticipated benefits for organizational and community resilience during the COVID‐19 pandemic. Our results illustrate a pathway for investment in system alignment efforts that build capacity within organizations and relationships between organizations to enhance resilience to crisis. Our findings suggest the usefulness of an integrated concept of organizational and community resilience that understands the resilience of systems of care as a vital resource for community resilience during crisis.

**What is known on this topic**

- System alignment efforts aim to coordinate among health care, social service, and public health organizations to achieve common goals.
- These efforts have been effective in improving population health and efficient use of resource, in part by better addressing social determinants of health.
- It is not known how system alignment processes evolve over time or may support resilience in response to challenges such as the COVID‐19 pandemic.

**What this study adds**

- Investments in aligning systems can build capabilities within organizations and relationships between organizations.
- Capabilities built by such investment, such adaptability, trust among partners, monitoring infrastructure and workforce, supported community resilience during the COVID‐19 pandemic.
- Investments in system alignment can have long‐term strategic benefits as communities face emerging health threats.

## 1 INTRODUCTION

In response to persistent challenges of health inequities and ineffective care, some regions have initiated efforts to align health care, social services, and public health organizations (that is, to coordinate to achieve common goals). System alignment seeks to remedy fragmented and siloed services and better coordinate health care with nonmedical and community‐based resources. The move toward alignment is being driven by the increased recognition that social determinants of health—such as income, housing, food, transportation, and more—profoundly shape health outcomes and spending.^1^ Alignment efforts have integrated screening for social needs in clinical settings, implemented systems to meet identified needs with social service resources, built integrated information systems across sectors, and established coordinated financing and governance structures.^2^ System alignment shows promise for promoting population health and efficient use of resources,^3^, ^4^, ^5^ but there is a lack of evidence on how system alignment processes evolve over time or may support resilience in response to challenges such as the COVID‐19 pandemic.

Resilience has been defined across individual, organization, and community levels and refers to the ability to adapt, maintain functioning, recover, and thrive in the face of adversity.^6^, ^7^, ^8^ Organizational resilience in health care defines a set of capacities enabling systems of care to adapt to change while maintaining high‐quality services.^9^, ^10^ Community resilience refers to the capacity to develop and engage community resources and to take collective action to influence the course of change.^7^, ^11^ Thus, community resilience to population health shocks such as the pandemic would be expected to depend on resilience within organizations providing essential resources, as well as how these organizations work together. While definitions of community resilience include availability of health care and other services,^12^ organizational and community resilience are rarely considered together.^13^ For this article, we use an integrated concept of organizational and community resilience, which understands organizational resilience in systems of health and social care as a crucial resource for community resilience during crises.

Community resilience in the context of pandemic involves supportive social networks, trusted information channels, access to knowledge and resources to mitigate spread, and civic engagement.^14^, ^15^ Community resilience during the COVID‐19 pandemic also requires the continued functioning of key economic and government institutions that provide essential goods and services. One crucial component of community resilience is resilience of health care delivery organizations, which entails the ability to respond quickly to surges in COVID‐19 cases and improvise solutions to unexpected problems while also maintaining high‐quality care for non‐COVID‐19 patients and the whole community.^10^, ^16^, ^17^ Additionally, community resilience during the pandemic requires the resilience of organizations beyond health care, including public health and social service organizations that address upstream factors and social determinants of health. Studies suggest that communities with greater alignment among health care, social services, and public health prior to the pandemic were able to mount stronger and more equitable responses.^18^ However, little is known about what contributes to the resilience of aligned systems or how particular aspects of these systems may serve as resources for community resilience. Therefore, we investigated how preexisting work to align health care, social services, and public health in one county in California was adapted during pandemic response and analyzed how prior alignment efforts may have supported resilience during crisis. We aimed to characterize resilience among county systems at the organizational level, and to consider how the resilience of these systems may support resilience of communities most vulnerable to public health crises.

## 2 METHODS

### 2.1 Study design

We chose an in‐depth qualitative study design to illuminate how prior work to align systems across health care, public health, and social services in Contra Costa County supported pandemic response. Methods were approved by the IRBs of UC Berkeley (Protocol 2020–08‐13,579) and Contra Costa Regional Medical Center.

### 2.2 Setting

Deep alignment among health care, social service, and public health systems remains relatively rare.^19^ We set our study in Contra Costa County, California, because it offers an example of advanced alignment, defined as organizations sharing systems across four specific areas: purpose, governance, financing, and data.^1^ The county health department, Contra Costa Health Services, encompasses the county's safety‐net hospital and a network of 10 primary care clinics, as well as public health services, and the Medicaid Managed Care plan that insures nearly 90% of the approximately 250,000 Medicaid beneficiaries in the county. Social services are administered by a sister unit of in the county government, the Employment and Human Services Department. While distinct units of county administration take responsibility for safety‐net health care, public health, Medicaid insurance, and social services, these units ultimately report to a common governance body, the county board of supervisors.

Beginning in 2016, a major ($200 million) 5‐year investment through California's Whole Person Care Medicaid waiver program^20^ supported the county to forge stronger links between health services and social services for the shared purpose of improving care for the county's Medicaid beneficiaries. Called CommunityConnect, this initiative provided additional shared financing for services across the health and social service sectors, as well as support for new data sharing infrastructure. At the core of CommunityConnect is a large‐scale social needs case management program, housed in the public health department, which coordinates care for individuals to meet their physical, behavioral health, and social needs. CommunityConnect employed about 120 case managers with a variety of backgrounds (nursing, social work, substance use counseling, mental health, community health work, and homeless services). Case management consisted of social needs screening, psychosocial support and motivational interviewing, and coordinating medical and social services. The digital infrastructure supported case management processes by providing real‐time data analytics about clients and facilitating workflows. The program served up to 12,000 patients at a time through a mix of in‐person and telephonic case management. Individuals were administratively enrolled through a risk model that prioritized people with a higher likelihood of acute care visits. Prior to the pandemic, a randomized study demonstrated that the program reduced inpatient hospitalizations by 11%.^21^

### 2.3 Data collection

At the time of our study, CommunityConnect represented the focal point of system alignment efforts in the county. Accordingly, we concentrated on CommunityConnect as the target of our data collection. We recruited participants through email and conducted semi‐structured phone interviews with a total of 31 informants, 14 of whom occupied managerial roles in public health, health care, or social services (including community‐based), and 17 of whom were social needs case managers who coordinated services across these sectors directly on behalf of patients. Participants were selected using purposive sampling to represent the range of professional expertise involved in this system alignment project (Table 1). Interviews were conducted by XX and XX between October 2020 and May 2021 and lasted 30–60 min.

**TABLE 1:** Interview sample by professional background.

Case managers	No. Interviewed
Public Health Nurse	5
Substance Use Counselor	2
Community Health Worker Specialist	4
Social Worker	1
Mental Health Clinical Specialist	3
Homeless Services Specialist	2
**Total**	**17**

### 2.4 Data analysis

All interviews were audio‐recorded, transcribed, and entered into NVivo analytic software (QSR International Pty Ltd. 2020, Version 12). An inductive‐deductive qualitative coding approach was used to systematically identify recurrent themes across transcripts.^22^ An initial list of deductive codes included features of the CommunityConnect program, implementation, and aspects of COVID‐19 response; inductive codes were added to capture new ideas. Then we finalized our code list and applied the final set of codes, double coding all transcripts.

To analyze how aligned systems were repurposed for pandemic response, we conducted a matrix analysis to identify data at the intersection of codes capturing key elements of system alignment (e.g., integration, data sharing, cross‐sector collaboration, organizational structure) and codes capturing key elements of pandemic response (e.g., support for COVID response, COVID‐related changes to system alignment, and adaptability). A matrix analysis approach is well suited to this research question as it explores the interactions and relationships between multiple dimensions of aligned system implementation and the COVID pandemic.^23^ Data at the intersections of these codes were categorized as capacities enabling aligned systems to be repurposed for pandemic response and defined as factors contributing to organizational resilience. From this analysis, we also identified ways aligned systems maintained or implemented essential services for the community as an indicator of organizational contributions to community resilience.

## 3 RESULTS

We identified four distinct components of the county's system alignment efforts that supported resilience during COVID‐19, including (a) an organizational culture of adaptability fostered during prior alignment efforts, (b) trusting relationships among organizations, (c) capacity to monitor population health of historically marginalized community members, and (d) frontline staff with flexible skills to support health and social care.

### 3.1 Organizational culture of adaptability

Over the 4 years preceding the pandemic, from 2016 to 2020, the county had rapidly developed system alignment and scaled up the CommunityConnect case management program at the core of its system alignment efforts, an ambitious undertaking housed in the public health department. A new organizational unit was created to deliver CommunityConnect, and that unit developed an organizational culture of adaptability and speed, encouraged by two factors. First, at the founding of CommunityConnect in 2017, county health leaders made a strategic decision to embed a dedicated continuous performance improvement team into the unit as part of a broader prioritization of efforts to spread quality improvement methodologies across the system. CommunityConnect's quality improvement team had five full‐time staff. As CommunityConnect ramped up services, adaptability and speed were traits seen as important to successfully developing a new, integrated care model in an accelerated timeframe. When the pandemic hit, the capacity and comfort with adaptability among CommunityConnect staff—particularly at the management level—closely matched the tasks required to scale up the emergency public health services of mass COVID‐19 testing and contact tracing. One administrator highlighted that established teams from CommunityConnect worked together on pandemic response, allowing the culture of adaptability (i.e., group norms) to transfer to these new assignments:

“All these people who have been getting CommunityConnect up and running were able to quickly shift and use their skills and use their already established teams in Connect, working together for this past year [of the pandemic], which gave them, I think, a real dexterity that wouldn't have been there at all, for us, at least, it we hadn't been through the last four years of CommunityConnect.” (Administrator 12).

Another administrator explained that specific demands of creating CommunityConnect—in terms of both speed and flexibility—had prepared the organization to work quickly, at scale, under uncertainty, as the pandemic response required:

“We had to hire, like, 100 people in several months to get CommunityConnect started. That was a real learning curve from CommunityConnect. We had already learned a lot about how you do things relatively quickly to bring things to scale.” (Administrator 3).

An administrator who had been involved in CommunityConnect and then managed elements of the public health department's pandemic response emphasized that CommunityConnect had recruited staff prepared to lead new programs amidst uncertainty, the same skills needed to contribute to pandemic response. Administrator 2 described this adaptable approach to work as “You just got to jump in and do it. Like, just get it started and you'll figure out, and you'll make mistakes, and you'll make tweaks.”

In sum, implementing system alignment before the pandemic demanded new ways of working at all levels of organizations. The efforts and innovations involved in system alignment cultivated a culture of adaptability that supported resilience during pandemic response.

### 3.2 Collaborative relationships among organizations

Efforts to align systems in the county over multiple years, with CommunityConnect as the most recent focal point, had established a history of collaboration among organizations providing health care, public health, and social services in the county. Positive experiences had built up habits of cross‐sector cooperation, which were seen as supportive of the COVID‐19 response. One interviewee reported that previous alignment work helped establish a strong working relationship between social service and public health departments, which enabled effective leadership during pandemic response:

“This is the difference that I see with this county as compared to [another county], that overall our relationship with public health is really positive and from my perspective, there's mutual trust. So, I just trusted and still do [the leader] to make the decisions that are right, for their larger team and for our community.” (Administrator 14).

Prior relationships formed between county health and social service departments through alignment efforts had been critical in adapting existing county services to support individuals experiencing homelessness to access housing as part of the COVID emergency response:

“I can't imagine if it was somebody that didn't have the connections like I do…because I already had those relationships built with our Health, Housing, and Homeless Division who really manages all those hotels and the logistics of the hotels, as well as our Healthcare for the Homeless team…” (Administrator 11).

Another participant explained that relationships and work processes developed between the public health department and social services departments allowed providers to streamline their work with common patients/clients during the pandemic:

“[The social services division] would send us lists of high risk patients that they were being mandated to reach out to by the state, and we were able to quickly cross reference and say, “Oh, well, 60% are on our list already, so we'll take care of it, so you guys only need to call, you know, these 400… so, that worked out great to save resources on both sides.” (Administrator 7).

Although trust, information sharing, and collaboration were generally described as positive across providers housed within the county government as well as community‐based organizations, community‐based stakeholders observed some dissatisfaction that resources for system alignment had initially been concentrated within the CommunityConnect unit, as opposed to being spread across a broader range of county and community‐based providers.

### 3.3 Capacity to monitor population health of historically marginalized community members

In addition to supplying teams poised to adapt to new challenges, the county's work to align systems also provided preexisting technical and management processes for population‐scale monitoring and outreach. These technical processes benefitted from an integrated data warehouse and team that linked records for a substantial proportion of the county's economically vulnerable population, integrating electronic health records, Medicaid claims, social service benefits, and data from other county‐administered systems. The data warehouse had been built through a multiyear process of working out data sharing agreements among relevant health care and social service organizations in the county.

Both the integrated data system and the management processes developed for its use were repurposed for COVID‐19 response. As one example, early in the pandemic, the data scientists who had created a predictive risk model to determine patients' eligibility for social needs case management in CommunityConnect also created a COVID‐19 vulnerability index to identify county residents at high risk of poor outcomes from COVID‐19. Individuals with high COVID‐19 vulnerability index scores were assigned to case managers who called to offer assistance with sheltering in place. The process leveraged the same EHR‐based workflows established prior to the pandemic to offer CommunityConnect case management. Interviewees explained how the processes that had been developed for CommunityConnect provided generalized capabilities to proactively reach out to vulnerable individuals during the pandemic, as one administrator noted:

“From the technical perspective, we had developed a system that you could throw anything at. You know? It doesn't matter if it's a system that supports patients with their social needs or as they need services to respond to COVID, or it could be, really, anything else.” (Administrator 6).

Another administrator emphasized the importance of having both technical systems and management processes built prior to the pandemic emergency, allowing these resources to be quickly deployed for new purposes:

“COVID, you know, caught us by surprise. And we had no time to… build all this infrastructure. So the fact that we had it all built, it didn't, you know, require ten days of us meeting—we were actually making phone calls and helping people with their food needs and transportation needs and trying to keep them safe at home… It only happened because of the infrastructure that was in place because of CommunityConnect.” (Administrator 4).

When the pandemic required the county's public health department to create new information systems to deliver COVID‐19 test results to county residents, they drew from existing system alignment processes associated with CommunityConnect:

“We [in the county public health department] do all of our [COVID‐19] testing, we do all of the notifications via text to the clients that come in, so they get their results instantaneously. As soon as we know it, they know it. So, we had to build all those systems. And I think CommunityConnect was the place that really showed us how we could do those things quickly and respond.” (Administrator 3).

Prior to the pandemic, CommunityConnect established a system in which case managers are notified when a community member considered to be high risk visited the emergency department or was admitted to the hospital, psychiatric emergency services, or jail. The system was designed to enable case managers to locate the patient at the relevant facility and coordinate a safe transition to the community. One case manager described how she used the notification system to locate a patient hospitalized for COVID‐19 and then used CommunityConnect resources to help him recover in the community:

“We get high risk notifications and so I was just informed he was in the hospital…I got in contact with him and…he was informed that he did have COVID. And he said just that, ‘I don't know where I'm going to go. I'm homeless, I'm living in my car. I don't know what to do.’ …Yeah, just one simple number and we got him housed, fed and everything else.” (Case Manager 24).

The preexisting systems for population health monitoring and integrated health and social care contributed to community resilience by offering the capacity to identify, locate, and support individuals at high risk of poor COVID‐19 outcomes.

### 3.4 Frontline staff with flexible skills to support health and social care

In the process of building the CommunityConnect case management program, the county had assembled a large staff of over 100 frontline case managers with training and experience to coordinate care across sectors. This workforce proved to be a particularly valuable resource for the county's COVID‐19 response. The CommunityConnect program continued to support thousands of low‐income individuals to navigate both health and social challenges throughout the pandemic. For many low‐income people in the county, the pandemic increased social needs.

“A lot of [the increased need] is housing, employment, and food. So, I think those are the three main things that people are worried about, especially if you're dealing with families. Especially if you're dealing with elders and they do not have the family support.” (Case Manager 25).

CommunityConnect's continued capacity to reach out and address integrated health and social needs during the pandemic was a crucial resource that could be leveraged to support community resilience.

“We had staff members who knew this type of work. They knew how to talk to individuals who were in a more vulnerable position. They knew how to ask them about social needs, and they knew what resources were available to meet those needs…” (Administrator 7).

In addition to directly addressing health and social needs, case managers provided social support to assist patients who were isolated and worried. As Case Manager 41 put it, “A lot of people just wanted to talk to me. They didn't want to be referred to Behavioral Health… they just wanted to talk to someone.”

Case managers also explained how their system navigation work changed to meet increased patient needs during the pandemic, while also helping patients navigate a landscape of changing assistance resources. Another case manager shared:

“During this pandemic it's been really helpful to just show up for patients… just providing a safe space for people to talk about what they're going through and then also that tangible, ‘Here's where you can go get food; Safeway is hiring right now’…resources that directly impact their wellbeing.” (Case Manager 04).

In addition to continuing social needs case management, the case managers were also redeployed to a variety of pandemic response roles, such as contract tracing for individuals with positive COVID‐19 test results. Interviewees reported that the case manager workforce embedded in the aligned systems was uniquely well suited for these emergency positions:

“Well, [CommunityConnect] gave us a bunch of people who could reach out to patients and address their individual needs. It gave us a workforce to help staff the new branches that we created as far as the COVID response. So, it was almost like the emergency workforce was right there until we can hire all these other temporary emergency workers, which just takes time…” (Administrator 1).

Interviewees reported that trust between frontline case managers and communities vulnerable to poor COVID‐19 outcomes, built through prior outreach and engagement efforts to meet community members' social needs, supported pandemic response.

“I think the trust that has been built with a large population that participated in our CommunityConnect program also led [them] to believe the public health department and our workers, gave us a certain trust that allowed us to respond more effectively and communicate more effectively with our county residents during the pandemic.” (Administrator 12).

Case managers who were redeployed as contact tracers often leveraged their experience to build trust between the public health agency and the community.

“In the beginning [of contact tracing], I think the hardest part was just building trust with the community that we were really trying to help them and not trying to get them in trouble or pry into their lives or tell them they were doing something wrong or getting them fired or telling them they had to be off work… I had a lot of case investigators that would say, ‘Nobody wants to talk to me. They won't give me their contacts.’… Helping people with housing and with food and with other resources along the way [during contact tracing]…they started to see that we really aren't trying to tell them they did anything wrong. We really just wanted to try to help them. And I think a huge part of that is that a lot of the people who came from CommunityConnect were deployed to do case investigation and naturally wanted to do a lot of those things anyways cause that's what they were trained to do.” (Case Manager 03).

Frontline staff with expertise in coordinating care across sectors—developed during system alignment efforts—contributed to community resilience by serving as a flexible emergency workforce and facilitating access to basic resources for community members vulnerable to poor COVID‐19 outcomes.

## 4 DISCUSSION

We documented multiple ways that prior investments in aligning systems within one county provided unanticipated benefits for organizational and community resilience, and facilitated emergency responses to the COVID‐19 pandemic. While prior work has identified key areas that communities need to consider when aligning across health care, social services, and public health (i.e., purpose, governance, financing and data),^1^, ^24^ our present analysis highlights opportunities that emerge as a result of alignment.

We identified four components of system alignment that were repurposed for pandemic response and supported organizational and community resilience (Table 2). These findings suggest that facilitators of organizational resilience (e.g., a culture of adaptability and trust among organizations) also supported community resilience during the pandemic. The county's organizational resilience enabled a public health response that included targeted outreach to individuals most at risk of poor COVID‐19 outcomes, continued provisioning of integrated health and social care for historically marginalized populations, and social support for isolated individuals. Further, frontline staff who were deployed for pandemic response drew from their experience working with lower income groups and the system capacity to address social needs to develop trust between the public health agency and the community.

**TABLE 2:** Components of system alignment contributing to organizational and community resilience.

Facilitators of resilience in alignment	Pre‐pandemic system alignment components	Contributions to organizational resilience during pandemic response	Contributions to community resilience during pandemic response
(1) Organizational culture of adaptability	Organizational unit focused on alignment Dedicated performance improvement team Experience with rapid scale up of new services	Rapid transition of staff roles Timely hiring of new roles Scale up of new pandemic‐related services	Robust pandemic response that included mass testing, contact tracing, and public health messaging
(2) Trusting relationships among organizations	Prior relationships established for system alignment	Effective leadership during crisis management Information sharing across organizations	Rapid implementation of shelter‐in‐place hotel program for people experiencing homelessness
(3) Capacity to monitor population health of historically marginalized community members	Integrated data warehouse and analytics team Management processes for proactive population outreach	Population health monitoring capacity repurposed for pandemic management	Proactive outreach to community members identified as high risk Assistance for patients with COVID who needed support with social needs
(4) Frontline staff with flexible skills to support health and social care	Case managers trained and experienced to work across sectors to deliver integrated services	Workforce available for pandemic deployment Case managers flexibly adapted skills	Delivered resources to meet needs of low‐income community members Trust between frontline case managers and community members aided pandemic response

One notable feature of this county's efforts at system alignment was the establishment of a large, new organizational unit (CommunityConnect) dedicated to linking care across health care, social services, and public health systems. Public administration theory suggests that stand‐alone integrator organizations can represent an advanced form of cross‐sector networking.^25^ Most of the benefits we document for pandemic response were promoted by the establishment of CommunityConnect as a distinct organizational unit. For example, the organizational culture of adaptability depended on CommunityConnect fostering a culture suited to its primary task among the approximately 150 staff. Establishing management processes for population monitoring and outreach and a cadre of frontline staff with skills to support health and social care were also facilitated by CommunityConnect existing as a distinct unit. These findings suggest that the establishment of a lead unit can play an important role in solidifying system alignment capabilities. Building a culture of adaptability could be especially important for public sector institutions, whose dedicated funding streams and regulatory requirements can limit flexibility.

Studies of organizational resilience find that resilience to crises and resilience to routine challenges typically rely on similar organizational capacities and processes.^8^, ^9^, ^26^ It may be helpful to think of processes to align systems across health care, social services, and public health as striving to enhance resilience to routine challenges, in the sense that alignment is forging new ways of working, making these efforts also applicable to discontinuous crisis response.

Although our study focuses on one county, findings may have theoretical generalizability to other settings.^27^ CommunityConnect was established through a Medicaid 1115 waiver program; many states have used related waivers to expand services related to social needs.^28^ Discussion of such waivers tends to focus on near‐term improvements in patient health and savings on acute care. Our research suggests that further attention should be paid to longer term strategic impacts on organizational capabilities and networks that support population health.

An important aim of aligning systems of health and social care is to better address unmet social needs caused by structures of inequality including poverty and structural racism.^29^ The social determinants of health that have underpinned persistent health inequalities prior to the pandemic also caused the deep inequalities observed in COVID‐19 outcomes. System alignment efforts may contribute to emergency response and community resilience by offering more robust systems for addressing social determinants of health and health care needs of people subject to structures of inequality. By more robustly meeting social needs, these efforts could also support community empowerment during crises and beyond.

Lastly, scholars of resilience have pointed out that the concept can inadvertently depoliticize crises, focusing attention on the capacity to adapt rather than on the political forces that cause or worsen crises, and unequally distribute their effects along lines of race, gender, class, and other structures of inequality.^30^ In the context of the COVID‐19 pandemic, this could mean attributing poor health outcomes to the lack of “capacities” among people who have been subject to structural violence, including from systems of health care, social services, and public health. As leaders seek to align and remake these systems, they should address the role of these systems in structural inequality as part of building organizational and community resilience.

Our study should be interpreted in light of several limitations. First, our data collection focused on identifying components of system alignment that were involved in pandemic response; we did not strive to identify components that were uninvolved in pandemic response or inhibited response. However, in exploring aligned systems' role in pandemic response, interviewees did not report any ways that alignment had inhibited response. Second, our study focused on alignment efforts that had been led by the county government and pandemic response activities organized by the county public health department. Although our interviews included individuals working in nonprofit community organizations as well, we did not comprehensively collect data on activities that did not involve the county administrative structures.

In conclusion, we have identified specific ways that long‐term, substantial investments in system alignment translated into enhanced response capabilities during the COVID‐19 pandemic. These findings provide tangible examples of benefits of aligned systems that may incentivize decision makers to invest in similar approaches to promote strengthened organizational and community resilience.
